# Non-weightbearing compared with weightbearing x-rays in hallux valgus decision-making

**DOI:** 10.1007/s00256-020-03441-9

**Published:** 2020-04-21

**Authors:** Andrzej Boszczyk, Sławomir Kwapisz, Maciej Kiciński, Bartłomiej Kordasiewicz, Henryk Liszka

**Affiliations:** 1 Department of Traumatology and Orthopaedics, Centre of Postgraduate Medical Education, Professor Adam Gruca Clinical Hospital, Konarskiego Str. 13, 05-400 Otwock, Poland; 2 Department of Orthopedics and Rehabilitation, University Hospital in Krakow, Krakow, Poland; 3 Department of Anatomy, University Hospital in Krakow, Krakow, Poland

**Keywords:** Bunion, Hallux valgus, Imaging, Weightbearing, Preoperative planning, Radiograph

## Abstract

**Objective:**

To report the effect of weightbearing x-ray imaging on clinical decisions in hallux valgus. Weightbearing (WB) x-ray is standard imaging for symptomatic hallux valgus (HV). In our clinical practice, often patients are presenting with non-weightbearing (NWB) x-rays. Repeated imaging requires additional radiation, justified only if expected to benefit patient’s treatment. In this study, the influence of WB status on radiological HV parameters and on clinical decisions was analyzed.

**Methods:**

In the dataset of WB and NWB x-rays, the hallux valgus (HVA) and intermetatarsal angle (IMA) were measured and differences analyzed. Clinical decisions for 10 x-ray pairs were studied among 40 respondents.

**Results:**

The WB and NWB HVA difference ranged − 16 to 16° (*p* < 0.001) and IMA − 3.4 to 5.8° (*p* < 0.001). In only 45% of cases, the decisions based on NWB and WB imaging were consistent (kappa (95% CI) = 30.0 (23.7–36.3)).

**Conclusions:**

Clinical decisions based on WB and NWB radiographs vary significantly. NWB films overestimate early and underestimate advanced HV deformity. Repeating radiographs is justified in patients presenting with NWB radiographs of symptomatic HV.

## Introduction

The standard imaging for a patient presenting with a symptomatic hallux valgus is weightbearing foot x-ray [1–3]. While the standard imaging is well established, it is sometimes not followed in clinical practice, where primary imaging is ordered in community clinics. In the case of the patient presenting with non-weightbearing x-rays, the surgeon is presented with a dilemma. Basing decision on non-weightbearing imaging may negatively influence the outcome, but repeating the imaging requires additional radiation. The repeated imaging, causing repeated radiation, is justified only if it is expected to benefit the patient’s treatment [4]. Majority of studies agree that radiological parameters of hallux valgus differ if measured in weightbearing and non-weightbearing imaging.

The results of available literature on significance of weightbearing imaging for clinical decision making are, however, contradictory, with some researchers reporting important influence [2] and others no influence of weightbearing on final surgical decisions [5].

In this study, we aimed to investigate (first) the influence of weightbearing status on radiological hallux valgus parameters and (second) on clinical decisions based on these radiographs.

The working hypothesis was that although the measured parameters would differ, the clinical decisions would remain unchanged.

## Method

The study was performed as a single-center retrospective case-control analysis of x-ray dataset. This is non-interventional study and formal consent was not required.

A set of 25 consecutive patients (38 feet) presenting between 2014 and 2017 were prospectively included in the study (demographic data in Table 1). The patients were included if they presented with symptomatic hallux valgus (diagnosis M20.1) and had non-weightbearing (NWB) x-rays performed no longer than 6 months prior to presentation of symptomatic foot available. As the NWB x-rays were brought from outside locations, their positioning was unstandardized. The exclusion criteria covered the following: previous surgery or signs of bony trauma at the foot, inadequate quality of non-weightbearing imaging. All patients had weightbearing (WB) x-ray of the foot which was performed with the film placed in a horizontal position and patient standing with weight distributed between both feet. The tube was angulated 15° posteriorly. Each foot was imaged separately with beam aimed at the second metatarsocuneiform joint. This way the dataset of 38 pairs of WB and NWB x-rays was collected.

**Table 1:** Summary of demographic data

	Total population	Females	Males
Number	25	22	3
Age mean (years)	47.5	47	51
Age range (years)	20–70	20–70	41–70

**Fig. 1:**
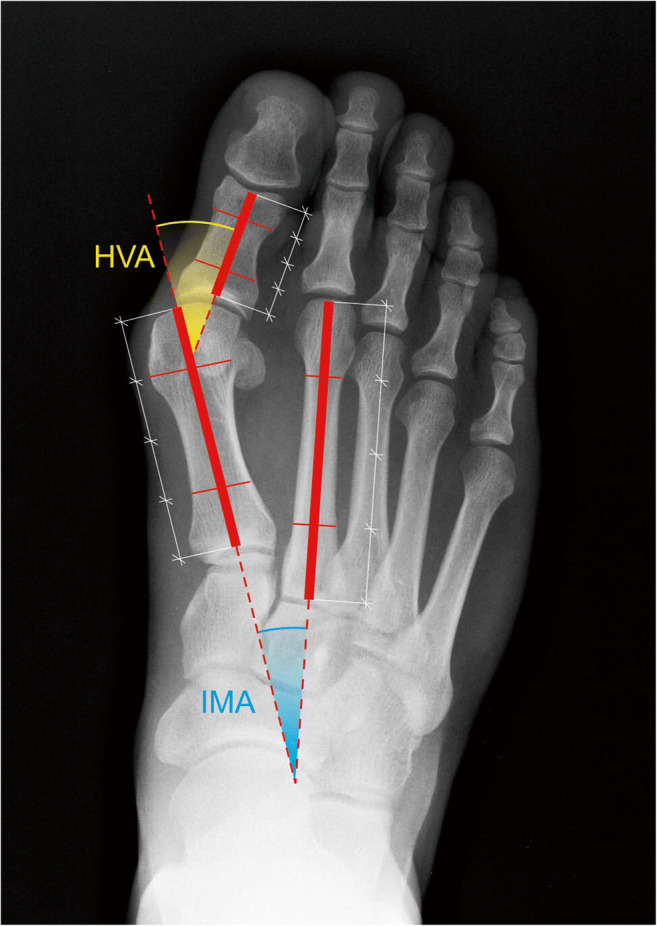
The method of HVA and IMA measurement—detailed description in text

The hallux valgus angle (HVA) and intermetatarsal angle (IMA) were measured by two orthopedic specialists in the whole set of 38 radiograph pairs [6]. To construct the first metatarsal axis, the length of first metatarsal was divided in four. Next, the points were marked in the middle of lines in between the proximal and second quarter as well as distal and third quarter of the metatarsal. The line constructed through these points represented first metatarsal axis. The axes of hallux proximal phalanx and of second metatarsal were constructed accordingly. The HVA was measured as an acute angle between the axis of first metatarsal and proximal phalanx and IMA as an acute angle between the axis of first metatarsal and second metatarsal (Fig. 1).

The difference between WB and NWB HVA, as well as WB and NWB IMA, was calculated.

For assessment of clinical decision-making from the set of 38 pairs of x-rays, the subset of 10 pairs was chosen. To represent the whole range of deformity, the pairs of x-rays were first arranged form minimal to maximal WBHVA; next, the first pair and every fourth pair were selected for the quiz. This way the subset of 10 pairs covering whole spectrum of deformity was selected. These 20 x-rays were presented in random order to orthopedic specialists and residents for assessment. The responders were asked to select the most appropriate procedure for each x-ray from a list. The responders did not know that the x-rays are paired. There were a total of 40 responders, with a mean of 12 years of experience in orthopedics (range 3–30 years); 9 responders declared themselves as foot and ankle surgeons and 18 responders are performing at least 20 and 8 responders over 70 hallux valgus corrections a year. To check intra-rater reliability of the clinical assessment, repeated assessments were performed by two experienced (> 70 surgeries/year) responders at least 1 week apart.

### Statistics

The normality of data for angular measurements was checked with the Smirnov-Kolomorgov test and the difference between weightbearing and non-weightbearing angular calculations assessed with Spearman’s correlation. The inter-rater and intra-rater reliability of angular measurements was assessed with Cronbach’s alpha by comparing two measurements of independent assessors and two measurements performed by single assessor at least 1 week apart. For further analysis, the results were averaged. The assessment of concordance of clinical decisions was performed with non-weighted Cohen’s kappa. The post hoc power analysis revealed that for 400 decisions (40 experts assessing 10 pairs) and expected inter-rater concordance of 70%, the 95% confidence interval lies between 50.2 and 89.9%.

## Results

### Angular measurements

**Fig. 2:**
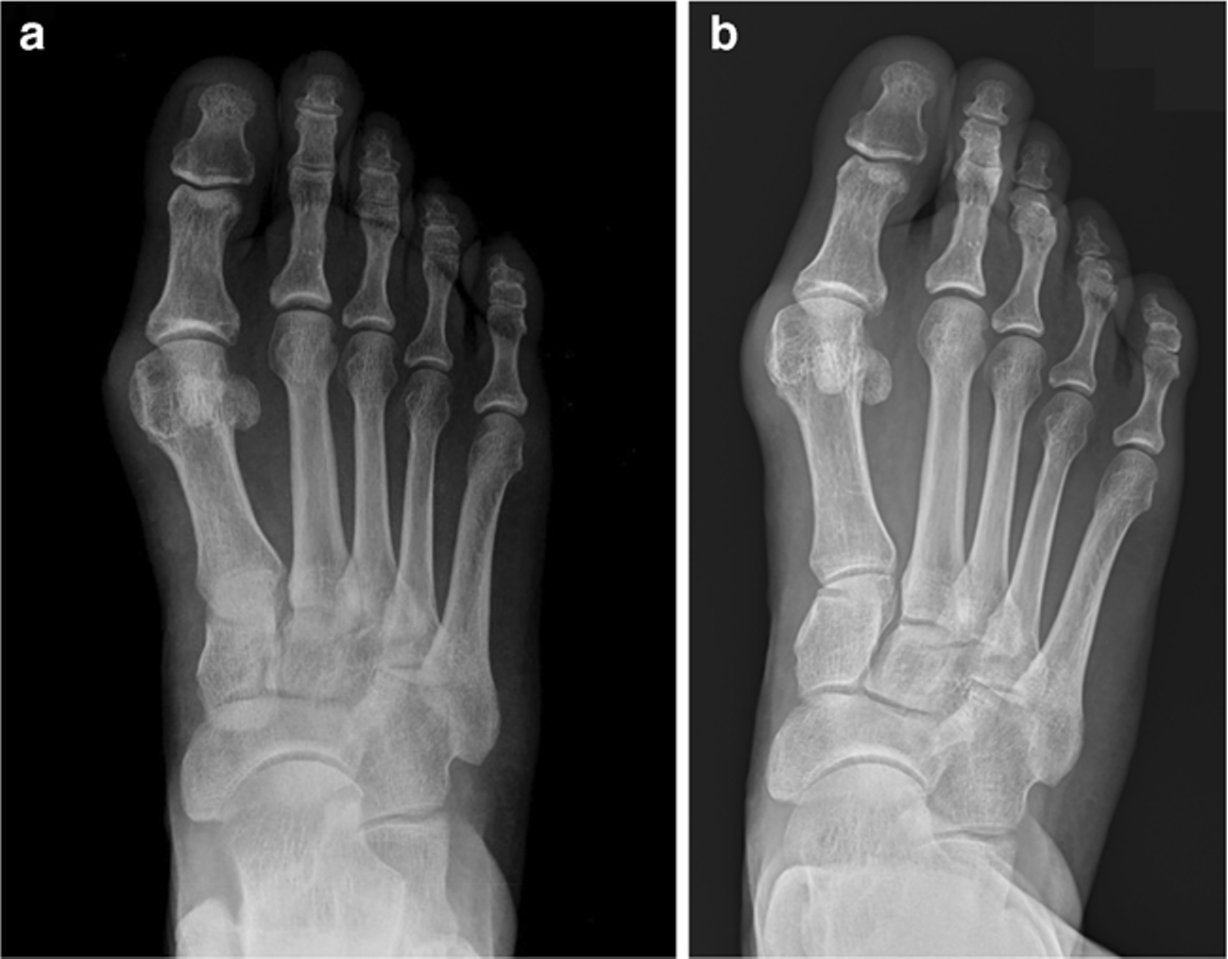
The non-weightbearing (**a**) and weightbearing (**b**) x-rays of the same patient. A 5° increase in HVA and 2.6° increase in IMA were observed

**Fig. 3:**
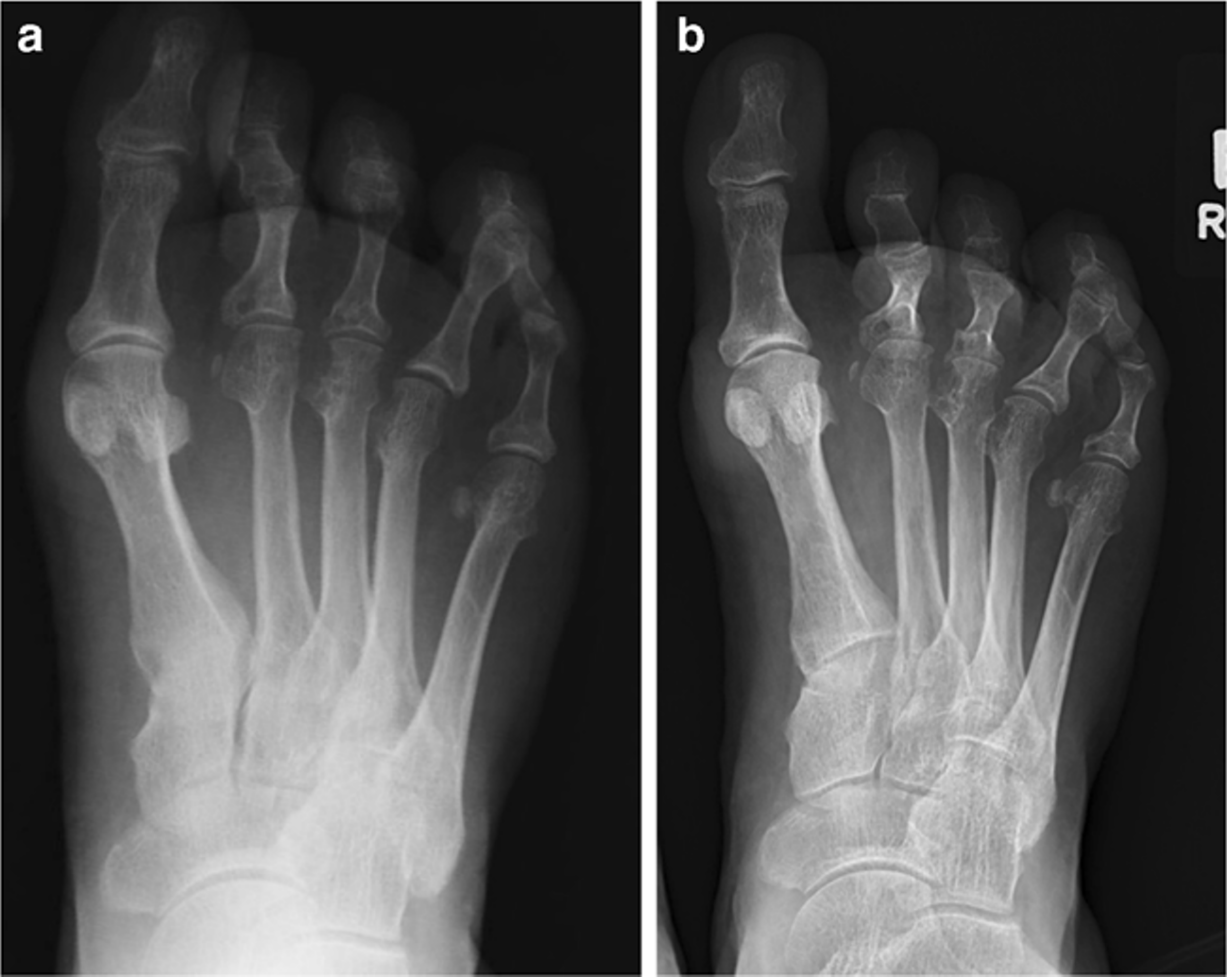
The non-weightbearing (**a**) and weightbearing (**b**) x-rays of the same patient. A 16° decrease in HVA and 3.4° decrease in IMA were observed

**Fig. 4:**
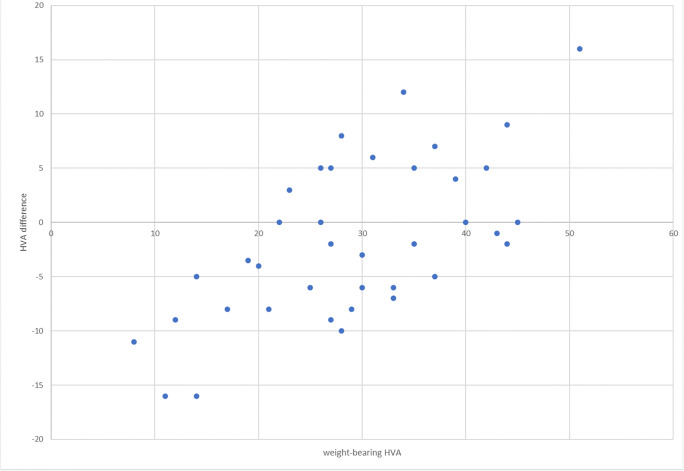
Difference in HVA depending on WBHVA. In smaller deformations, the HVA reduces under load while in larger deformations, the HVA increases under load

The HVA in the non-weightbearing averaged 30.8° (range 19–46) and in weightbearing 29.1° (8–51), respectively. The difference between weightbearing and non-weightbearing HVA averaged − 1.64 (negative value representing reduction in HVA under weightbearing) ranging from − 16 to 16. For smaller WBHVA, there was a reduction in value observed under load, while for larger WBHVA, the opposite was observed (Figs. 2 and 3). The difference in HVA in relation to WBHVA is statistically significant (*p* < 0.001) and is presented in Fig. 4.

**Fig. 5:**
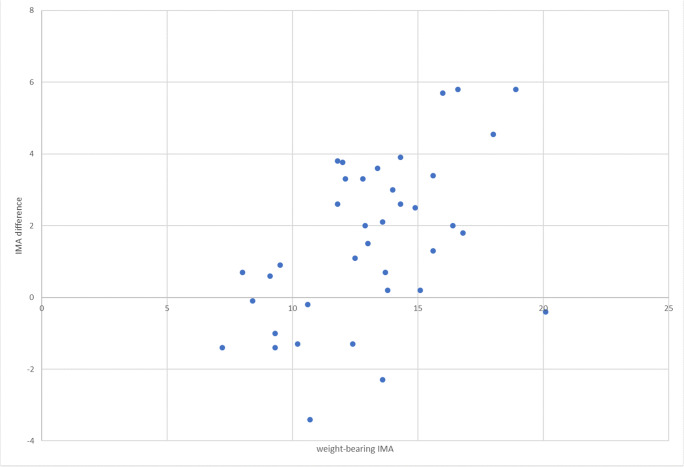
Difference in IMA depending on WBIMA. In smaller deformations, the IMA reduces under load while in larger deformations, the IMA increases under load

The IMA in the non-weightbearing cohort averaged 11.5° (range 7.3–20.5) and in weightbearing group 13.1 (7.2–20.1), respectively. The difference between weightbearing and non-weightbearing IMA averaged 1.57 (positive value representing elevation of IMA under weightbearing) ranging from − 3.4 to 5.8. The difference in IMA in relation to WBIMA is statistically significant (*p* < 0.001) and is presented in Fig. 5.

The alpha for inter-rater repeatability was 0.98 for HVA and 0.92 for IMA measurement and intra-rater test revealed alpha of 0.97 for HVA and 0.92 for IMA—all translating into excellent consistency.

### Clinical decisions

The 40 clinicians assessed the 10 pairs of x-rays resulting in 400 pairs of decisions (800 decisions). In 14 cases, the answers were excluded due to atypical answers, such as an option to correct the hindfoot. One responder produced 9 of these atypical pairs, so his/her report has been excluded from analysis.

**Fig. 6:**
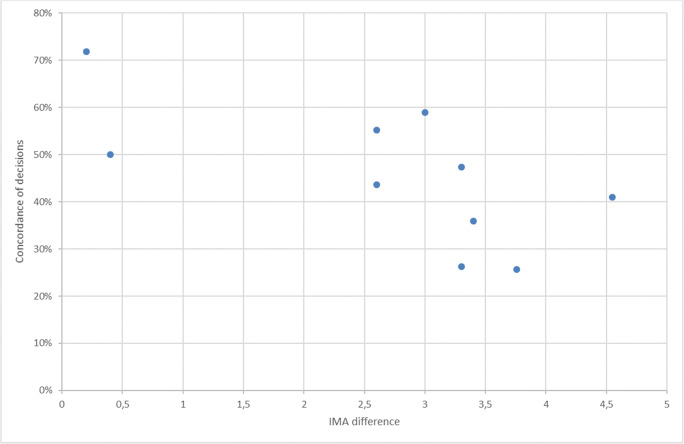
Concordance of clinical decisions based on IMA difference between WB and NWB imaging. The bigger the IMA difference within x-ray pair, the more variability in clinical decisions

In 175 of 386 pairs (45%) of the cases, the decisions based on non-weightbearing imaging were consistent with weightbearing-based decisions, and in 55% of cases, the surgical qualification has changed (kappa (95% CI) = 30.0 (23.7–36.3)). The concordance of assigned treatments ranged from 26 to 72% for each patient. The concordance level was significantly reversely correlated with the IM difference between WB and NWB images (Fig. 6). What is of special interest in the case representing minimal deformity (WBHVA = 8° and WBIMA = 12.8°), nonoperative treatment was assigned three times based on non-weightbearing x-rays and 6 times based on weightbearing x-rays.

The agreement was lower for a subgroup with mild deformity—38%—in comparison with the subgroup with more severe deformity—52%.

When analyzing in details the differences between clinical decisions based on weightbearing and non-weightbearing radiographs, in 79%, the indication was for more proximal correction (for example shaft osteotomy instead of distal osteotomy) when WB radiographs were used.

The concordance level ranged from 20 to 70% for each responder. This percentage was higher in the subgroup of foot and ankle specialists (average = 54%) than surgeons not declaring this specialization (average = 43%).

The assessment of intra-rater reliability revealed that the clinical decisions in 83% of cases were identical.

## Discussion

The standard imaging for a patient presenting with a symptomatic hallux valgus is a weightbearing x-ray [1–3, 7, 8]. In the clinical reality, the patient often presents with non-weightbearing x-rays and the surgeon presented with a dilemma. Our current standard is repeating imaging with weightbearing. The repeated imaging, however, causing repeated radiation, is justified only if it is expected to benefit the patient’s treatment. The aim of this study was to investigate influence of radiographic technique on hallux valgus parameters as well as clinical decisions based on WB and NWB radiographs.

The main finding of our study is that clinical decisions made after assessment of weightbearing and non-weightbearing imaging vary significantly. The reason for observed differences in decisions is the difference in radiographic angular measurements important for surgical planning in hallux valgus.

Our study showed that the impact of weightbearing on HVA and IMA is variable and depends on the actual WBHVA and WBIMA of the patient. While this variability has been observed before, it has not been linked to the hallux valgus severity [9]. Previous studies reported simple increase in IMA with weightbearing [2, 10]. To the contrary, in our observation, the non-weightbearing x-rays tended to overestimate the early deformity, while they underestimated the severity of the advanced hallux valgus. This observation, we believe, illustrates progressive joint instability in hallux valgus deformity progression [11].

What is most important from clinical point of view is observation that decisions for more than half of the patients have changed after assessment of weightbearing imaging. This contrasts the findings of the study of Burg who found no difference in decisions nor in angles measured between WB and NWB films [5]. What is noticeable, however, is the fact that in Burg’s study only experienced surgeons (> 50 cases per year) assessed the x-rays. This can partly explain their results, as in our study this population also produced more consistent decisions.

Similar to our observations, the study assessing clinical decision-making with regard to foot malpositioning also revealed changes in clinical decisions [1].

What is noticeable is the fact that the surgeons assessing the same x-rays on two separate occasions (intra-rater) achieved concordance of their decisions in only 83% of cases. This reflects subjectivity of decisions in hallux valgus, as well as overlapping indications for a given procedure.

The high female overrepresentation may be viewed as a potential weakness of the study. In our opinion, however, it reflects the reality of hallux valgus surgery. We believe that artificial equalization of the sex ratio would not be reasonable for the study.

The fact that clinical decisions were based on 10 pairs of x-rays may also be viewed as a weakness of the study. The cases, however, were specifically selected to cover whole spectrum of deformity and were assessed by 40 surgeons with variable level of experience resulting in 400 decisions for analysis and such practice has been successfully used in other studies of similar construction [12].

The weakness of the study is the fact that the decisions had to be made without clinical information and that there could be no standardization of NWB x-rays as they were performed outside our institution. Also the assumption that the more experienced responders were unaware of the fact that some of the films were performed without weightbearing may not be true.

## Conclusions

The clinical decisions based on weightbearing and non-weightbearing radiographs vary significantly.

Weightbearing films are crucial for assessment of hallux valgus deformity.

Repeating radiographs is justified in patients presenting with non-weightbearing radiographs of symptomatic hallux valgus.

In early hallux valgus, non-weightbearing films tend to overestimate the deformity while in advanced deformation they tend to underestimate it.
